# The Use of Embedded IMU Insoles to Assess Gait Parameters: A Validation and Test-Retest Reliability Study

**DOI:** 10.3390/s23198155

**Published:** 2023-09-28

**Authors:** Louis Riglet, Fabien Nicol, Audrey Leonard, Nicolas Eby, Lauranne Claquesin, Baptiste Orliac, Paul Ornetti, Davy Laroche, Mathieu Gueugnon

**Affiliations:** 1 CHU Dijon-Bourgogne, Centre d’Investigation Clinique, Module Plurithématique, Plateforme d’Investigation Technologique, 21000 Dijon, France; 2 INSERM, CIC 1432, Module Plurithématique, Plateforme d’Investigation Technologique, 21000 Dijon, France; 3 Zhortech SAS, 54000 Nancy, France; 4 INSERM, UMR1093-CAPS, Université Bourgogne Franche-Comté, UFR des Sciences du Sport, 21000 Dijon, France; 5 Rheumatology Department, CHU Dijon-Bourgogne, 21000 Dijon, France

**Keywords:** gait analysis, insole, validation, test-retest reliability, criterion validity, 3D motion analysis

## Abstract

Wireless wearable insoles are interesting tools to collect gait parameters during daily life activities. However, studies have to be performed specifically for each type of insoles on a big data set to validate the measurement in ecological situations. This study aims to assess the criterion validity and test-retest reliability of gait parameters from wearable insoles compared to motion capture system. Gait of 30 healthy participants was recorded using DSPro^®^ insoles and a motion capture system during overground and treadmill walking at three different speeds. Criterion validity and test-retest reliability of spatio-temporal parameters were estimated with an intraclass correlation coefficient (ICC). For both systems, reliability was found higher than 0.70 for all variables (*p* < 0.001) except for minimum toe clearance (ICC < 0.50) with motion capture system during overground walking. Regardless of speed and condition of walking, Speed, Cadence, Stride Length, Stride Time and Stance Time variables were validated (ICC > 0.90; *p* < 0.001). During walking on treadmill, loading time was not validated during slow speed (ICC < 0.70). This study highlights good criterion validity and test-retest reliability of spatiotemporal gait parameters measurement using wearable insoles and opens a new possibility to improve care management of patients using clinical gait analysis in daily life activities.

## 1. Introduction

Quantified gait analysis is a widespread tool used to accurately measure human locomotion outcomes, particularly, spatiotemporal and kinematic parameters [1]. Using 3 dimensional (3D) motion capture system, gait analysis is considered as the gold standard for gait characterization with reliable and accurate measurements of movement [2,3,4]. Used in multiple fields of medicine, this system has notably allowed to improve and help the clinical diagnostic for patients with musculoskeletal and neurological pathologies [2,5]. However, the principal’s disadvantages of this system are its long operation time, dedicated space requirement, technical expertise requirement and high cost [2]. Moreover, standard clinical gait analysis produces a broad range of data, needing expertise to analyze and produce comprehensible report to the patient.

To overcome these disadvantages, new gait analysis systems have been developed to measure similar biomechanical parameters in gait research. Using incorporating software and algorithms, Inertial Measurement Units (IMUs) allow to measure spatiotemporal and kinematic parameters of each segment on which they are positioned [6]. Currently, IMUs are the most used sensors devices in multiple fields as diagnosing gait disabilities [7,8,9], control mechanics of prostheses [10], etc. This wearable system can be used easily in daily life activities in various conditions [11]. However, IMUs have a trend to shift during measurement impacting their accuracy, that is one of their biggest limits.

To record the movements and orientations of the foot, IMU can be fixed on or under the foot. Based on algorithms, the IMU data helps to recognize walking steps and to calculate spatiotemporal parameters of locomotion. Not limited to the gait laboratory or the clinical set up, the use of insoles emerges in a lot of fields like disease detection, rehabilitation and seems to grow every day [11]. Moreover, many studies have been carried out to confirm the relevance of the measurement performed using sensorized insoles [12,13,14,15,16,17,18,19,20,21]. However, the principal variables studied allowing this validation are limited: step counting and cadence [15,16], vertical force [17,18], or partial gait parameters [12,19,20].

Formerly called PODOSmart^®^, DSPro^®^ insoles, were developed and have the advantage of being able to collect data during different gait tasks. Compared to a gait analysis system, recent studies have highlighted the good accuracy [22] and test-retest reliability [23] of these insoles. However, the study carried out by Ziagkas et al. [22] was performed on a small number of participants (n = 11), all men, and a small number of analyzed gait cycles (two per participants). As mentioned by the authors, this is not sufficient to reflect the natural variability of human gait as well as the heterogeneity of the gait speeds. Moreover, the 3D marker set used in this study is also not the one which offers the best calculation of kinematics, gait cycle events, that could alter the quality of the spatiotemporal computed outcomes. Therefore, before using these wearable insoles in a clinical context for out- and in-patients, it is necessary to validate their reliability and validity without these limitations.

To our knowledge, no validation of this insole has been performed on big data set of gait cycles in healthy subjects. Moreover, with a clinical potential use, this insole validation seems fundamental to improve care management of patients. In this context, the purposes of this study were to assess the test-retest reliability and validity of gait parameters from DSPro^®^ insoles compared to gait parameters obtained from a 3D motion capture system considered as the gold standard measurement system.

## 2. Materials and Methods

### 2.1. Population

A total of 30 participants were included in this monocentric protocol from October 2021 to February 2022. The inclusion criteria were: healthy subjects, older than 18 years old, who was able to understand simple orders and instructions for locomotion and who lived within a maximum radius of 50 km from the investigation site. The major exclusion criteria were persons who were not affiliated to national health insurance, subject to a legal protection measure, unable to express consent, presented a disarticulated hip, diseases or disabilities that have an impact on walking. All participants included in this study provided informed oral consent. The study protocol was approved by the local institutional ethics committee and authorized by the French National Agency for Drug Safety. This study has been published in Clinical Trial registration (reference NCT05104645).

### 2.2. Procedure & Materials

**Figure 1:**
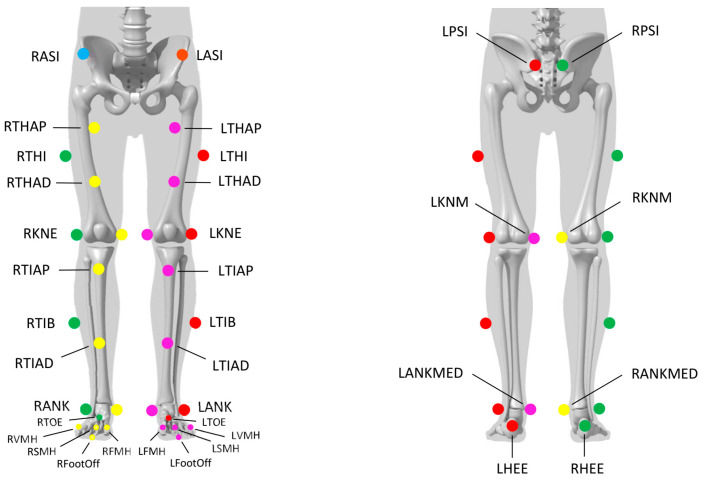
Position of the markers on the anatomical landmarks following the Conventional Gait Model (version 2.5).

**Figure 2:**
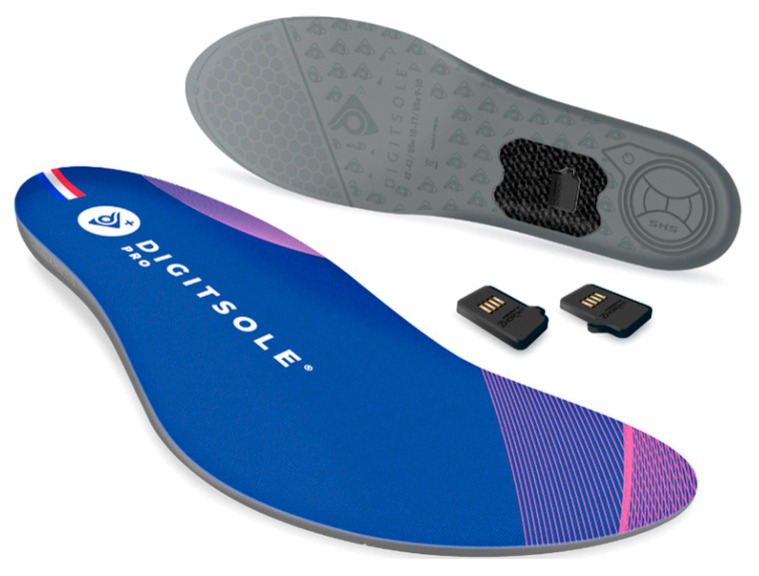
DSPro^®^ insole device.

Experimentations were conducted in Marey Institute, from the laboratory INSERM U1093 “Cognition, Action and Sensorimotor Plasticity” (Dijon, France) and carried out two visits per participant (second visit seven days after the first one). Visits were performed at the same day of the week and at similar time slots to restrict gait day-fluctuations [24]. All participants were equipped with 32 reflective cutaneous markers positioned following the Conventional Gait Model (version 2.5) lower-body markers set [25,26] (Figure 1). One marker was added (R/L ToeOff) at the extremity of the shoes, close to the hallux. The 3D position of the markers was recorded using 18 optoelectronic cameras operating at a sampling rate of 100 Hz (11 VERO and 7 MX-T10 cameras, Vicon System^®^, Oxford, UK; 100 Hz) using Nexus software (2.12.1 version). Two forceplates were embedded in the floor to record ground reaction forces (AMTI^®^, USA; 1000 Hz) and one numerical camera was positioned in the sagittal plane. Moreover, each participant was equipped with identical walking shoes (Ekiden One, Kalenji^®^) in which insole was fitted (DSPro^®^ featuring Zhortech^®^ algorithms; Digitsole SAS, Nancy, France) (Figure 2). A sensor was integrated into each insole, which was itself chosen, as well as the corresponding pair of shoes, according to the size announced by the subject and considering its good congruence with the real size of the participant’s foot. Each sensor embeds accelerometers, gyroscopes, and magnetometers to measure linear accelerations and angular velocities in the 3 planes of space. Each sensor records movements with sampling frequency of 104 Hz.

Two gait conditions were performed: on a walkway (10-m) and on a treadmill (EF 1800, Medical Developpement). Moreover, before each condition, a subject calibration was performed in order to calibrate IMUs and consisted for the participant to maintain a steady position for 3 s. To not disturb the locomotor pattern of participants, the speed was chosen as naturally as possible to be in the most ecological and comfortable situations. In the first condition, participant was asked to walk along a straight line at their comfortable and self-selected speed (Figure S1 in Supplementary Materials), then at a slower speed and finally at a faster walking speed spontaneously selected. For each gait speed, participants performed at least 5 back and worth. In the treadmill condition, participants also walked at comfortable, slow and fast walking speed. The comfortable walking speed was selected by the volunteer after a 2-min familiarization trial and set using the treadmill dashboard (constant speed). The slow speed was then calculated by removing 1 km/h from the comfortable speed and the fast speed was calculated by adding 2 km/h. Each speed condition was performed for 2 min, followed by 1 min of recovery.

### 2.3. Data Analysis

Both systems (inertial units in sole and motion capture) were synchronized by an analogical button to trig the beginning of each participant’s trial. Data from both devices were separately collected and stored during the execution of 10 trials for each participant on the walkway for each gait speed and during 2 min for each treadmill conditions.

For motion capture, markers from pelvis to foot were used in order to reconstruct embedded coordinate systems associated to each rigid body segment (pelvis, femur, tibia and foot) defining then a complete 3D model of the lower limb. Marker trajectories were interpolated with Woltring polynomial and then filtered with a low pass zero phase shift Butterworth filter with a respective cut off frequency of 6 Hz. Similarly, ground reaction forces were filtered with a low pass zero phase shift Butterworth filter with a respective cut off frequency of 50 Hz [27]. The gait events were then computed using a method proposed by O’Connor et al. [28] based on the position of the foot velocity algorithm. This method is based on the creation of a new trajectory, representing the foot center by calculating the midpoint of the heel and toe marker locations. The maximal vertical foot velocity corresponds to the Toe-off (TO) and the minimum as the Heel-strike (HS). Then, gait cycle was defined and the time between two successive HS of the same leg. Two other gait events were computed: Flat Foot In (FFI) and Flat Foot Out (FFO) corresponding to the first frame in which the foot is flat on the ground and the last frame in which the foot is still flat on the ground, respectively. FFI was computed using the height of the toe marker (R/LTOE) being lower than a threshold estimated by the minimal height of the TOE marker plus 5% of the range of motion of the toe in the beginning of the gait cycle (until 15% of gait cycle [29]). FFO was found when the height of the heel marker (R/LHEE) was superior to the following threshold estimated by the minimal height of the HEE marker plus 5% of the range of motion of the HEE marker between FFI and TO frames. For insoles, sensors have allowed to segment the walk into different specific events of the normal walk, based on HS and TO. FFI and FFO were also computed and correspond to the sample in which the toe touches the ground after HS and the sample in which the heel takes off the ground after HS. Gait cycle was considered as synchronized between the two systems if the time difference between initial foot strike was lower than 0.35 s. All post-processing was performed using custom-made scripts with Python (3.9 version) and Matlab (MathWorks^®^; R2021a version). Examples of typical raw data obtained with both systems (positions of RHEE and RTOE markers for motion capture and normalized acceleration and angular velocity for insoles) are illustrated in Figure S2.

For motion capture, the definition of parameters computed and extracted for each gait speed and each condition is detailed in Table S1.

### 2.4. Statistical Analysis

Test-retest reliability was estimated by comparing each parameter from each system between the two sessions. The computation of the intra-class correlation (ICC and its 95% CI), based on a mean-rating, absolute agreement, 2-way mixed-effects model (as suggested by Koo & Li [30]) for each system between the two sessions was therefore made on the average parameter for each gait type (overground and on treadmill), each session, each speed condition and each side (left or right gait cycle). ICC, confidence interval (IC) of 95% and *p*-value (*p*) were extracted. Participant was the target variable (30) and Session was the rater variable (2).

Criterion validity was computed to estimate if the spatio-temporal parameters from embedded insoles agree with those from the gold standard (i.e., Vicon). It was obtained with an intraclass correlation coefficient (ICC), based on a mean-rating, consistency, 2-way mixed-effects model [30], independently of the session and the gait side for the two systems. These two analyses were performed on the mean of each parameter collected for each speed condition (independently of the gait cycle and of the session). Moreover, Bland-Altman analysis are also performed on each gait parameter for each condition.

Based on Koo & Li [30], ICC was considered as poor, moderate, good and excellent for values lower than 0.5, between 0.5 and 0.75, between 0.75 and 0.9 and higher than 0.9, respectively. Systems were defined as reliable and valid if ICC was higher than 0.50. Correlations were considered as statistically significant for *p* < 0.003 (0.05 divided by the number of variables (15)) [31]. All these analyses were performed separately in terms of gait type and speed condition and were calculated with Stata software (17.0 version).

## 3. Results

### 3.1. Population Characteristics

The sample of the validation study consisted of a healthy adult population including 14 females and 16 males without diagnosed gait disorders. Their age ranged from 21 to 36 years (mean 27.6 ± 5.2 years) for the female group and from 21 to 42 years (mean 28.3 ± 6.1 years) for the male group. The female mean height was 165.2 ± 5.3 cm (17 cm range) and it was 180.3 ± 5.3 cm (18 cm range) for male, and the female mean weight was 61.0 ± 9.2 kg (30 kg range) when the male mean weight was 74.4 ± 8.6 kg (30 kg range). Finally, the female mean shoe size was 39.2 ± 1.8 (6 size range) and the male mean shoe size was 43.4 ± 1.5 (5 size range).

### 3.2. Synchronization of Gait Cycle

For overground walking, 4824 gait cycles were recorded between the three gait speed conditions. Thus, 4807 gait cycles were matched corresponding to 99.6% of our data set: 1625 cycles for comfortable, 1399 cycles for fast and 1789 cycles for slow speed.

For walking on treadmill, 18 159 gait cycles were recorded between the three gait speed conditions on treadmill. Thus, with the same synchronized condition as overground walking, 18 048 gait cycles were matched corresponding to 99.4% of our data set: 5924 cycles for comfortable, 7064 cycles for fast and 5060 cycles for slow speed.

### 3.3. Test-Retest Reliability

Mean and STD of gait parameters for both sessions with motion capture system and insole device for overground walking, and ICC values were presented in Table 1 and Table 2, respectively. For motion capture system, ICC values were higher than 0.75 for all gait parameters except for speed (comfortable: ICC = 0.724, *p* < 0.001) and for minimum toe clearance (fast: ICC = 0.708, *p* = 0.001). Moreover, non-significative correlation was found for minimum toe clearance for slow (ICC = 0.336, *p* = 0.144) and comfortable (ICC = 0.480, *p* = 0.046) speed. For insole device, ICC values were higher than 0.75 for all gait parameters except for Speed variable for fast speed (ICC = 0.748, *p* < 0.001).

Mean and STD of gait parameters for both sessions with motion capture and insoles for walking on treadmill, and ICC values were presented in Table 3 and Table 4, respectively. High ICC values were found for all gait parameters for both system (ICC > 0.80, *p* < 0.001) except for Plantar Flexion Foot In for motion capture at comfortable (ICC = 0.744, *p* < 0.001) and slow speed (ICC = 0.737, *p* < 0.001).

### 3.4. Criterion Validity

For all speed walking, mean and standard deviation of gait parameters for both systems, and ICC values were presented for overground and treadmill walking in Table 5 and Table 6, respectively. Bland-Altman plots for all gait parameters during each velocity condition in overground and on the treadmill are detailed in Figure S3 and S4, respectively. 

For overground condition, the mean bias of temporal parameters (stride time, stance time, loading time, flat foot time, propulsion time, double support time, and swing time) for insoles (compared to motion capture gold standard) were found close to zero seconds independently of the value of the average measurement. Increasing walking speed (slow, comfortable, or fast conditions) did not change the bias value. The mean bias for spatial parameters (speed, cadence, and stride length) was also close to zero but the heterogeneity of the error seems to increase with speed (increasing from slow, comfortable to fast conditions). For swing width and toe clearance bias seems linearly related to the increase of the average measurement (positively for swing width and negatively for toe clearance). Angle parameters showed a mean bias of about 3° with a similar standard deviation between the different speeds.

ICC results revealed that all parameters during overground walking were significantly correlated between systems for the three different speeds with ICC values from 0.673 to 0.999 (*p* < 0.002). Speed, cadence, stride length, stride time, stance time, swing time, and foot progression angle (7/15) showed excellent intraclass correlations (ICC > 0.90, *p* < 0.001). Swing width, double support time and plantar flexion foot in (3/15) exhibit at least a good correlation (ICC > 0.75, *p* < 0.001) for all gait speeds. Loading time, flat foot time, propulsion time, plantar foot out and minimum toe clearance (5/15) exhibited from moderate to excellent correlation in function of the gait speed condition (ICC > 0.50, *p* < 0.002).

During treadmill walking, Bland-Altman analyses showed the same results as in overground walking with a low bias for temporal parameters (with an increase in the heterogeneity of the bias with the increase in speed conditions for stride time) and identical observations for other parameters. ICC results revealed that all parameters were significantly correlated between systems for the three different speeds with ICC values from 0.678 to 0.999 (*p* < 0.002) except for the loading time condition (ICC = 0.562, *p* = 0.015) for slow speed. Speed, cadence, stride length, stride time, stance time, swing time, and foot progression angle (7/15) showed an excellent correlation (ICC > 0.90, *p* < 0.001). Double support time, swing width, plantar flexion foot In and Out, and minimum toe clearance (5/15) exhibit at least a good correlation (ICC > 0.75, *p* < 0.001) for all gait speeds. Flat foot time and propulsion time (2/15) exhibited from moderate to excellent correlation in function of the gait speed condition (ICC > 0.50, *p* < 0.002). Loading time showed a moderate and good correlation for comfortable and fast speed conditions, respectively.

## 4. Discussion

The main objective of this study was to assess the criterion validity and the test-retest reliability of gait parameters from DSPro^®^ insoles in healthy subjects compared to gait parameters obtained with motion capture system. Gait analysis data were collected from 30 healthy participants during two sessions of overground and treadmill walking. Our results demonstrate for the first time the relevance of the gait analysis obtained with DsPro^®^ insoles during walking for different gait speeds in overground as well as in a treadmill. In addition, the use of three different speeds during overground and treadmill walking constitutes a new dataset and allows a more accurate and complete validation of these insoles.

First, the detection of the gait events (FO and FS) and so, the gait cycle, allows to evaluate more than 99% of the totality of gait cycles available and help to obtain a large data set (more than 4800 and 18,000 cycles recorded for overground and treadmill walking, respectively). By ensuring the good synchronization of the two systems, the cycles measured by the IMUs and by the Motion Capture were compared in order to evaluate the detection capacity of the IMUs walking instants compared to the reference. This is essential in order to measure and calculate the various temporal parameters, but also to define the instants at which the calculations of the signals for the temporal and kinematic parameters must begin. The high number of gait cycles in the 3 different speeds allow to take into account the natural variability of human gait speed which could not be measured in previous study [22]. The following results are therefore stronger, relevant and more representative of the human gait.

Test-retest reliability was measured to quantify the consistency of parameters measurements. For the two-walking condition, results revealed a good test-retest reliability of the measurements taken with the inertial units (ICC > 0.75) for all gait parameters (except for Speed variable at fast speed overground walking, ICC = 0.748). It can be explained by the fact that insoles can be easily installed under the same conditions during each new session, which guarantees the monitoring and comparison of results over time. Moreover, these test-retest reliability results are in agreement with the results presented by Loukovitis et al. [23]. In fact, the authors have highlighted ICC values varying from 0.802 to 0.997. However, in the Loukovitis et al. [23] study, the number of gait cycle analyzed is weaker compared to ours that could decrease the heterogeneity of the data and therefore improve the results. Additionally, minimum toe clearance measured by motion capture was not reliable during overground walking condition for slow and comfortable speed (no significant ICC values). This could be associated with the known difficulty to estimate this parameter with motion system analysis. However, we used a validated method previously used [32]. Our mean values of this parameter for motion capture and insoles are moreover in agreement with the literature [22,23]. For other kind of IMU, Homan et al. showed good ICC values since all parameters were above 0.836, except stride velocity (ICC = 0.763) and double support (ICC = 0.703).

Furthermore, the assessment of criterion validity also revealed positive results. Bland-Altman plots show in most of the cases, a low heterogeneity of the bias between the two measurements carried out by the two systems with a mean bias close to 0 for spatiotemporal parameters in overground and treadmill conditions. Moreover, a 3-degree bias was found for angle parameters and as to be considered during measurement. However, as the bias was found to be constant across conditions, it does not impair its use when comparing subjects with the same system. Further measurements should be done to ensure the usability of the insole with another system in order to control the bias. In addition, the bias seemed to be linearly related to the average measurement for swing width and minimum toe clearance without effect of the walking speed conditions (slow, comfortable and fast). This point has to be of particular attention, since the bias increases or decreases in function of the measurement value. Further study has to focus on this point in order to complete these results. These observations are confirmed by the ICC results with good to excellent ICC values for most of the outcomes. More specifically, the main spatio-temporal parameters (speed, cadence, stride length, stride time, stance time, swing time, swing width, double support time, foot progression angle and plantar flexion foot in) exhibited all a correlation higher than 0.75 showing a good validity of these parameters obtained by the insoles, and so independently of the gait speed and of the gait conditions (overground walking and treadmill). The ICC results for the temporal parameters are in agreement with the results presented by Ziagkas et al. [22] (ICC varied from 0.546 to 0.999). However, loading time was not validated in treadmill walking condition for slow speed (*p* > 0.003). These results are comparable with those of other validation studies of IMU positioned at the foot against a 3D motion analysis system. Schwameder et al. [33] have highlighted a good Pearson correlation coefficient (r = [0.936;0.987]) for spatiotemporal parameters during normal walking as well as during limping gait. More recently, Jakob et al. [34] also showed a high correlation coefficient for spatiotemporal parameters (ICC = [0.964;0.986]) by IMUs and motion capture. Interestingly, they also revealed high correlation for kinematics parameters (ICC = [0.822;0.903]). However, Homan et al. [35] have highlighted that the spatiotemporal parameters had a higher ICC between the two systems than the kinematic parameters, with excellent ICC for the swing speed (ICC = 0.994), stride length (ICC = 0.979), peak angle velocity (ICC = 0.961), flat-foot duration (ICC = 0.961), cycle duration (ICC = 0.932), cadence (ICC = 0.930) and stride velocity (ICC = 0.927).

This study validates the use of theses insoles during overground and treadmill walking and highlights in particular its significant interest for clinic fields. The use of such tool for the follow-up of healthy people practising leisure walking seems particularly adapted in order to follow and improve the gait. In another context, these low-cost insoles could allow clinical centres to perform gait analysis without a motion capture system. For instance, health professionals could not have access to motion capture system since it needed a dedicated space requirement, technical expertise requirement and high cost [2]. Thereby, gait parameters can notably be obtained for pathological patients during walking on a treadmill and help to improve patient care management with objective and more specific parameters than speed or duration.

While this study has some limitations (our sample is composed by young participants), the results are robust since they rely on a significant amount of gait cycle, performed on overground and on treadmill and with different gait speed. Altogether, this could compensate this limitation and offer a first step for using these insoles in normal gait in different contexts. To extend its use in more specific contexts like clinical or running context, further investigations must be performed. Notably, future research aims to explore the use of this insole in patients suffering from the sequelae of stroke, with the ultimate aim of facilitating the evaluation of locomotion in these patients. Indeed, the evaluation of locomotor capacities is a key element in the rehabilitation of patients, making it possible to quantify the autonomy of patients and the impact of rehabilitation exercises on the reduction of stroke-related disorders.

## 5. Conclusions

In conclusion, this study confirms and validates the good validity and test-retest reliability of gait parameters measurement using DSPro^®^ insoles compared to motion analysis system. While wearable device does not allow to obtain a global assessment of participants locomotion, especially kinematics, these embedded insoles seem to become a new pertinent tool for easiest quantified gait analysis. While the test-retest reliability seems high, further investigations have to be performed to validate its use, in particular for clinical gait analysis as well as for sport analysis such as running. Notably with its low cost, user-friendly and wearable aspect, these devices offer new opportunities to improve care management of patients with altered locomotor pattern (neurologic, orthopedic, etc.).

## Figures and Tables

**Table 1:** Mean and STD of gait parameters for both sessions with motion capture for overground walking. ICC and its 95% CI were also indicated. Significant relations were illustrated as follows: * = *p* < 0.001. Non-significant relations were illustrated in italics.

	OVERGROUND WALKING—MOTION CAPTURE								
	*Slow Speed*			*Comfortable Speed*			*Fast Speed*		
	Mean ± STD		ICC [95% CI]	Mean ± STD		ICC [95% CI]	Mean ± STD		ICC [95% CI]
	*Session 1*	*Session 2*		*Session 1*	*Session 2*		*Session 1*	*Session 2*	
Speed (m.s^−1^)	0.95 ± 0.15	1.02 ± 0.17	0.906 [0.702;0.962] *	1.40 ± 0.11	1.45 ± 0.10	0.724 [0.413;0.870] *	1.75 ± 0.15	1.82 ± 0.14	0.767 [0.487;0.891] *
Cadence (step.min^−1^)	45.48 ± 4.65	46.73 ± 4.28	0.926 [0.827;0.966] *	56.20 ± 2.21	57.13 ± 2.24	0.810 [0.577;0.913] *	62.30 ± 3.37	63.52 ± 3.99	0.873 [0.724;0.940] *
Stride Length (m)	1.25 ± 0.10	1.30 ± 0.12	0.892 [0.670;0.956] *	1.50 ± 0.09	1.52 ± 0.08	0.888 [0.761;0.948] *	1.69 ± 0.10	1.72 ± 0.10	0.917 [0.801;0.963] *
Stride Time (s)	1.35 ± 0.16	1.31 ± 0.13	0.924 [0.820;0.966] *	1.07 ± 0.04	1.05 ± 0.04	0.810 [0.578;0.913] *	0.97 ± 0.05	0.95 ± 0.06	0.900 [0.765;0.953] *
Stance Time (s)	0.86 ± 0.11	0.83 ± 0.10	0.912 [0.784;0.961] *	0.65 ± 0.03	0.64 ± 0.03	0.775 [0.506;0.896] *	0.57 ± 0.04	0.56 ± 0.04	0.888 [0.728;0.950] *
Loading Time (s)	0.14 ± 0.02	0.13 ± 0.02	0.905 [0.753;0.959] *	0.11 ± 0.01	0.10 ± 0.01	0.788 [0.380;0.914] *	0.10 ± 0.01	0.09 ± 0.01	0.847 [0.573;0.936] *
Flat Foot Time (s)	0.43 ± 0.07	0.41 ± 0.07	0.940 [0.867;0.972] *	0.29 ± 0.04	0.29 ± 0.04	0.888 [0.762;0.948] *	0.23 ± 0.04	0.22 ± 0.03	0.896 [0.781;0.951] *
Propulsion Time (s)	0.30 ± 0.04	0.28 ± 0.03	0.883 [0.739;0.946] *	0.25 ± 0.03	0.25 ± 0.03	0.963 [0.900;0.984] *	0.24 ± 0.03	0.24 ± 0.03	0.936 [0.866;0.969] *
Double Support Time (s)	0.37 ± 0.08	0.35 ± 0.07	0.886 [0.703;0.951] *	0.23 ± 0.02	0.22 ± 0.02	0.766 [0.497;0.891] *	0.17 ± 0.03	0.16 ± 0.03	0.825 [0.723;0.952] *
Swing Time (s)	0.89 ± 0.04	0.48 ± 0.03	0.948 [0.889;0.975] *	0.42 ± 0.01	0.42 ± 0.01	0.900 [0.774;0.953] *	0.40 ± 0.02	0.39 ± 0.02	0.925 [0.842;0.964] *
Foot Progression Angle (°)	−1.02 ± 2.71	−1.69 ± 2.57	0.930 [0.846;0.968] *	−1.61 ± 2.43	−2.32 ± 2.42	0.903 [0.786;0.955] *	−0.93 ± 2.64	−1.72 ± 2.30	0.912 [0.795;0.960] *
Swing Width (cm)	2.92 ± 1.01	3.15 ± 1.06	0.942 [0.848;0.975] *	2.73 ± 0.91	2.87 ± 1.00	0.936 [0.854;0.971] *	2.94 ± 1.07	2.90 ± 1.06	0.953 [0.902;0.978] *
Plantar Flexion Foot In (°)	18.82 ± 2.04	19.34 ± 2.23	0.856 [0.700;0.931] *	21.35 ± 2.30	21.41 ± 2.52	0.917 [0.823;0.961] *	24.25 ± 2.44	24.48 ± 2.80	0.926 [0.846;0.965] *
Plantar Flexion Foot Out (°)	−60.19 ± 4.15	−61.47 ± 4.16	0.885 [0.747;0.946] *	−65.14 ± 3.83	−66.06 ± 3.74	0.905 [0.795;0.956] *	−68.93 ± 3.92	−69.04 ± 4.35	0.948 [0.891;0.976] *
Minimum Toe Clearance (cm)	1.50 ± 0.42	1.51 ± 0.41	*0.336 [−0.429;0.688]*	1.62 ± 0.47	1.70 ± 0.52	*0.480 [−0.115;0.757]*	1.68 ± 0.69	1.82 ± 0.66	0.708 [0.369;0.860] *

**Table 2:** Mean and STD of gait parameters for both sessions with insole for overground walking. ICC and its 95% CI were also indicated. Significant relations were illustrated as follows: * = *p* < 0.001.

	OVERGROUND WALKING—INSOLES								
	*Slow Speed*			*Comfortable Speed*			*Fast Speed*		
	Mean ± STD		ICC [95% CI]	Mean ± STD		ICC [95% CI]	Mean ± STD		ICC [95% CI]
	*Session 1*	*Session 2*		*Session 1*	*Session 2*		*Session 1*	*Session 2*	
Speed (m.s^−1^)	0.98 ± 0.15	1.05 ± 0.17	0.898 [0.681;0.959] *	1.43 ± 0.11	1.48 ± 0.10	0.756 [0.444;0.889] *	1.77 ± 0.15	1.86 ± 0.15	0.748 [0.405;0.887] *
Cadence (step.min^−1^)	45.17 ± 4.55	46.44 ± 4.42	0.921 [0.817;0.964] *	55.86 ± 2.27	57.15 ± 2.27	0.806 [0.481;0.918] *	61.78 ± 3.48	63.55 ± 4.01	0.866 [0.648;0.942] *
Stride Length (m)	1.30 ± 0.10	1.35 ± 0.12	0.878 [0.641;0.950] *	1.53 ± 0.10	1.56 ± 0.09	0.907 [0.789;0.957] *	1.73 ± 0.11	1.76 ± 0.11	0.896 [0.770;0.952] *
Stride Time (s)	1.35 ± 0.15	1.31 ± 0.13	0.921 [0.819;0.964] *	1.08 ± 0.04	1.05 ± 0.04	0.813 [0.491;0.921] *	0.97 ± 0.06	0.95 ± 0.06	0.890 [0.703;0.953] *
Stance Time (s)	0.86 ± 0.12	0.82 ± 0.10	0.908 [0.784;0.959] *	0.64 ± 0.03	0.43 ± 0.03	0.782 [0.488;0.902] *	0.56 ± 0.04	0.055 ± 0.04	0.907 [0.727;0.961] *
Loading Time (s)	0.12 ± 0.02	0.11 ± 0.01	0.886 [0.758;0.946] *	0.10 ± 0.01	0.10 ± 0.01	0.912 [0.760;0.963] *	0.09 ± 0.01	0.08 ± 0.01	0.788 [0.526;0.902] *
Flat Foot Time (s)	0.45 ± 0.08	0.42 ± 0.08	0.917 [0.798;0.963] *	0.29 ± 0.02	0.29 ± 0.03	0.838 [0.658;0.924] *	0.24 ± 0.03	0.23 ± 0.03	0.949 [0.881;0.977] *
Propulsion Time (s)	0.29 ± 0.03	0.29 ± 0.02	0.845 [0.677;0.926] *	0.25 ± 0.02	0.25 ± 0.02	0.919 [0.824;0.962] *	0.23 ± 0.02	0.23 ± 0.02	0.927 [0.848;0.965] *
Double Support Time (s)	0.36 ± 0.08	0.33 ± 0.06	0.882 [0.716;0.947] *	0.21 ± 0.02	0.21 ± 0.02	0.800 [0.534;0.896] *	0.16 ± 0.02	0.15 ± 0.03	0.892 [0.762;0.950] *
Swing Time (s)	0.50 ± 0.04	0.49 ± 0.04	0.943 [0.881;0.973] *	0.43 ± 0.02	0.42 ± 0.02	0.882 [0.736;0.946] *	0.41 ± 0.02	0.41 ± 0.02	0.896 [0.746;0.954] *
Foot Progression Angle (°)	−5.58 ± 2.69	−5.50 ± 2.67	0.974 [0.946;0.988] *	−5.53 ± 2.57	−5.52 ± 0.23	0.984 [0.966;0.993] *	−4.59 ± 2.39	−4.62 ± 2.56	0.982 [0.963;0.992] *
Swing Width (cm)	3.29 ± 1.67	3.52 ± 1.71	0.934 [0.873;0.971] *	3.06 ± 1.53	3.26 ± 1.53	0.937 [0.867;0.970] *	3.22 ± 1.65	3.30 ± 1.52	0.943 [0.881;0.973] *
Plantar Flexion Foot In (°)	20.11 ± 2.20	20.89 ± 2.65	0.893 [0.761;0.950] *	23.54 ± 2.09	23.84 ± 2.23	0.919 [0.829;0.962] *	25.20 ± 2.54	25.74 ± 2.38	0.929 [0.847;0.967] *
Plantar Flexion Foot Out (°)	−56.68 ± 3.73	−58.08 ± 4.87	0.902 [0.769;0.956] *	−61.55 ± 3.41	−61.83 ± 4.17	0.917 [0.825;0.961] *	−65.86 ± 3.42	−66.87 ± 4.31	0.924 [0.935;0.964] *
Minimum Toe Clearance (cm)	1.76 ± 0.63	1.58 ± 0.55	0.883 [0.720;0.947] *	1.76 ± 0.74	1.53 ± 0.59	0.812 [0.574;0.919] *	1.56 ± 0.66	1.42 ± 0.56	0.788 [0.554;0.900] *

**Table 3:** Mean and STD of gait parameters for both sessions with motion capture for walking on treadmill. ICC and its 95% CI were also indicated. Significant relations were illustrated as follows: * = *p* < 0.001.

	WALKING ON TREADMILL—MOTION CAPTURE								
	*Slow Speed*			*Comfortable Speed*			*Fast Speed*		
	Mean ± STD		ICC [95% CI]	Mean ± STD		ICC [95% CI]	Mean ± STD		ICC [95% CI]
	*Session 1*	*Session 2*		*Session 1*	*Session 2*		*Session 1*	*Session 2*	
Speed (m.s^−1^)	0.82 ± 0.11	0.82 ± 0.11	0.999 [0.999;0.999] *	1.08 ± 0.11	1.08 ± 0.11	0.999 [0.997;0.999] *	1.62 ± 0.11	1.62 ± 0.11	0.999 [0.998;0.999] *
Cadence (step.min^−1^)	42.90 ±3.51	42.81 ± 3.33	0.944 [0.881;0.973] *	50.78 ± 2.76	50.08 ± 3.10	0.910 [0.807;0.957] *	60.39 ± 3.03	60.47 ± 2.58	0.975 [0.948;0.988] *
Stride Length (m)	1.08 ± 0.10	1.09 ± 0.09	0.951 [0.898;0.977] *	1.23 ± 0.09	1.24 ± 0.09	0.956 [0.906;0.979] *	1.57 ± 0.10	1.56 ± 0.09	0.984 [0.965;0.992] *
Stride Time (s)	1.42 ± 0.12	1.42 ± 0.11	0.936 [0.865;0.969] *	1.19 ± 0.06	1.21 ± 0.07	0.909 [0.802;0.957] *	1.00 ± 0.05	1.00 ± 0.04	0.972 [0.942;0.987] *
Stance Time (s)	0.93 ± 0.09	0.93 ± 0.08	0.957 [0.910;0.980] *	0.75 ± 0.05	0.77 ± 0.05	0.927 [0.830;0.967] *	0.60 ± 0.03	0.60 ± 0.03	0.975 [0.947;0.988] *
Loading Time (s)	0.16 ± 0.02	0.16 ± 0.02	0.915 [0.822;0.959] *	0.13 ± 0.01	0.13 ± 0.01	0.858 [0.682;0.934] *	0.11 ± 0.01	0.11 ± 0.01	0.891 [0.773;0.948] *
Flat Foot Time (s)	0.48 ± 0.06	0.51 ± 0.06	0.934 [0.752;0.975] *	0.36 ± 0.05	0.39 ± 0.04	0.856 [0.271;0.952] *	0.26 ± 0.04	0.27 ± 0.03	0.913 [0.619;0.969] *
Propulsion Time (s)	0.29 ± 0.04	0.27 ± 0.04	0.894 [0.705;0.955] *	0.26 ± 0.03	0.24 ± 0.03	0.854 [0.428;0.947] *	0.24 ± 0.03	0.22 ± 0.03	0.901 [0.398;0.968] *
Double Support Time (s)	0.44 ± 0.06	0.45 ± 0.06	0.980 [0.957;0.991] *	0.32 ± 0.03	0.33 ± 0.03	0.954 [0.866;0.981] *	0.21 ± 0.02	0.21 ± 0.02	0.971 [0.940;0.986] *
Swing Time (s)	0.49 ± 0.04	0.48 ± 0.03	0.877 [0.742;0.942] *	0.43 ± 0.02	0.44 ± 0.02	0.877 [0.745;0.947] *	0.40 ± 0.02	0.39 ± 0.02	0.963 [0.921;0.982] *
Foot Progression Angle (°)	−1.23 ± 2.61	−2.21 ± 2.80	0.916 [0.784;0.963] *	−1.41 ± 2.62	−2.19 ± 2.90	0.924 [0.830;0.965] *	−1.48 ± 2.41	−2.12 ± 2.44	0.906 [0.799;0.955] *
Swing Width (cm)	3.04 ± 1.15	3.21 ± 1.32	0.902 [0.796;0.953] *	2.79 ± 0.80	3.15 ± 0.97	0.850 [0.573;0.937] *	2.90 ± 0.79	3.03 ± 0.83	0.934 [0.869;0.972] *
Plantar Flexion Foot In (°)	16.32 ± 2.08	16.72 ± 1.84	0.737 [0.452;0.875] *	17.60 ± 2.45	18.75 ± 2.10	0.744 [0.436;0.881] *	22.77 ± 2.58	22.92 ± 2.45	0.902 [0.795;0.954] *
Plantar Flexion Foot Out (°)	−56.97 ± 3.71	−57.11 ± 4.07	0.940 [0.873;0.971] *	−62.47 ± 3.72	−62.61 ± 3.62	0.929 [0.850;0.966] *	−69.56 ± 3.49	−69.38 ± 3.88	0.948 [0.890;0.975] *
Minimum Toe Clearance (cm)	1.73 ± 0.55	1.77 ± 0.44	0.855 [0.695;0.931] *	1.99 ± 0.65	2.00 ± 0.55	0.879 [0.744;0.942] *	1.77 ± 0.65	1.80 ± 0.56	0.833 [0.648;0.921] *

**Table 4:** Mean and STD of gait parameters for both sessions with insole for walking on treadmill. ICC and its 95% CI were also indicated. Significant relations were illustrated as follows: * = *p* < 0.001.

	WALKING ON TREADMILL—INSOLES								
	*Slow Speed*			*Comfortable Speed*			*Fast Speed*		
	Mean ± STD		ICC [95% CI]	Mean ± STD		ICC [95% CI]	Mean ± STD		ICC [95% CI]
	*Session 1*	*Session 2*		*Session 1*	*Session 2*		*Session 1*	*Session 2*	
Speed (m.s^−1^)	0.84 ± 0.11	0.84 ± 0.11	0.999 [0.998;0.999] *	1.11 ± 0.11	1.11 ± 0.11	0.997 [0.997;0.999] *	1.63 ± 0.11	1.63 ± 0.10	0.998 [0.995;0.999] *
Cadence (step.min^−1^)	42.89 ± 3.50	42.83 ± 3.34	0.944 [0.881;0.973] *	50.73 ± 2.75	50.01 ± 3.08	0.908 [0.803;0.957] *	59.89 ± 3.02	60.32 ± 2.47	0.952 [0.899;0.977] *
Stride Length (m)	1.17 ± 0.10	1.18 ± 0.10	0.937 [0.868;0.970] *	1.31 ± 0.09	1.33 ± 0.09	0.939 [0.864;0.972] *	1.65 ± 0.11	1.63 ± 0.09	0.921 [0.832;0.963] *
Stride Time (s)	1.42 ± 0.12	1.42 ± 0.11	0.935 [0.864;0.969] *	1.19 ± 0.06	1.21 ± 0.07	0.908 [0.800;0.957] *	1.01 ± 0.05	1.00 ± 0.04	0.929 [0.851;0.966] *
Stance Time (s)	0.92 ± 0.09	0.92 ± 0.08	0.960 [0.916;0.981] *	0.75 ± 0.05	0.76 ± 0.05	0.935 [0.852;0.970] *	0.60 ± 0.04	0.60 ± 0.03	0.975 [0.978;0.988] *
Loading Time (s)	0.11 ± 0.01	0.11 ± 0.01	0.935 [0.863;0.969] *	0.10 ± 0.01	0.10 ± 0.01	0.907 [0.792;0.957] *	0.09 ± 0.01	0.09 ± 0.01	0.941 [0.881;0.973] *
Flat Foot Time (s)	0.51 ± 0.07	0.51 ± 0.06	0.962 [0.921;0.982] *	0.36 ± 0.04	0.37 ± 0.04	0.921 [0.772;0.967] *	0.25 ± 0.03	0.26 ± 0.03	0.954 [0.905;0.978] *
Propulsion Time (s)	0.30 ± 0.02	0.30 ± 0.02	0.894 [0.779;0.950] *	0.28 ± 0.02	0.28 ± 0.02	0.919 [0.827;0.961] *	0.26 ± 0.02	0.25 ± 0.02	0.912 [0.816;0.958] *
Double Support Time (s)	0.42 ± 0.06	0.43 ± 0.06	0.983 [0.964;0.992] *	0.30 ± 0.04	0.31 ± 0.04	0.961 [0.907;0.982] *	0.20 ± 0.03	0.20 ± 0.02	0.973 [0.943;0.987] *
Swing Time (s)	0.50 ± 0.04	0.49 ± 0.04	0.878 [0.745;0.942] *	0.44 ± 0.02	0.45 ± 0.02	0.877 [0.744;0.941] *	0.40 ± 0.02	0.40 ± 0.02	0.961 [0.920;0.982] *
Foot Progression Angle (°)	−5.85 ± 2.94	−6.40 ± 2.96	0.971 [0.930;0.987] *	−5.45 ± 2.91	−5.74 ± 3.08	0.983 [0.965;0.992] *	−4.92 ± 2.49	−5.00 ± 2.50	0.984 [0.966;0.992] *
Swing Width (cm)	3.14 ± 1.98	3.53 ± 2.16	0.917 [0.812;0.961] *	3.00 ± 1.33	3.61 ± 1.55	0.872 [0.556;0.951] *	2.85 ± 0.99	3.09 ± 0.95	0.940 [0.867;0.972] *
Plantar Flexion Foot In (°)	16.52 ± 2.19	19.43 ± 2.19	0.953 [0.900;0.978] *	18.86 ± 2.55	19.43 ± 2.43	0.903 [0.794;0.954] *	23.98 ± 2.22	23.58 ± 1.80	0.917 [0.826;0.960] *
Plantar Flexion Foot Out (°)	−54.56 ± 3.79	−54.76 ± 4.69	0.866 [0.718;0.936] *	−59.25 ± 3.58	−59. 65 ± 4.30	0.883 [0.759;0.944] *	-67.77 ± 3.56	−67.13 ± 3.24	0.916 [0.824;0.960] *
Minimum Toe Clearance (cm)	1.54 ± 0.58	1.50 ± 0.59	0.929 [0.852;0.966] *	1.63 ± 0.70	1.55 ± 0.65	0.895 [0.782;0.950] *	1.25 ± 0.55	1.18 ± 0.55	0.926 [0.846;0.965] *

**Table 5:** Mean and STD of gait parameters for both systems for overground walking. ICC and its 95% CI were also indicated. Significant relations were illustrated as follows: * = *p* < 0.001 and + = *p* < 0.002.

	OVERGROUND WALKING								
	*Slow Speed*			*Comfortable Speed*			*Fast Speed*		
	Mean ± STD		ICC [95% CI]	Mean ± STD		ICC [95% CI]	Mean ± STD		ICC [95% CI]
	*Motion Capture*	*Insoles*		*Motion Capture*	*Insoles*		*Motion Capture*	*Insoles*	
Speed (m.s^−1^)	0.98 ± 0.20	1.01 ± 0.19	0.999 [0.998;0.999] *	1.42 ± 0.10	1.45 ± 0.10	0.997 [0.992;0.998] *	1.79 ± 0.14	1.81 ± 0.14	0.993 [0.985;0.997] *
Cadence (step.min^−1^)	46.11 ± 5.38	45.80 ± 5.34	0.999 [0.998;0.999] *	56.61 ± 2.00	56.44 ± 2.07	0.992 [0.983;0.996] *	62.90 ± 3.63	62.67 ± 3.67	0.992 [0.983;0.996] *
Stride Length (m)	1.28 ± 0.13	1.32 ± 0.14	0.996 [0.992;0.998] *	1.51 ± 0.08	1.54 ± 0.09	0.996 [0.992;0.998] *	1.70 ± 0.10	1.74 ± 0.10	0.984 [0.966;0.992] *
Stride Time (s)	1.32 ± 0.19	1.33 ± 0.18	0.999 [0.999;0.999] *	1.06 ± 0.04	1.07 ± 0.04	0.994 [0.988;0.997] *	0.96 ± 0.05	0.96 ± 0.06	0.992 [0.984;0.996] *
Stance Time (s)	0.84 ± 0.14	0.84 ± 0.14	0.998 [0.997;0.999] *	0.64 ± 0.03	0.64 ± 0.03	0.981 [0.960;0.991] *	0.56 ± 0.04	0.55 ± 0.04	0.991 [0.981;0.996] *
Loading Time (s)	0.13 ± 0.03	0.12 ± 0.02	0.714 [0.390;0.864] +	0.10 ± 0.01	0.10 ± 0.01	0.679 [0.326;0.847] +	0.10 ± 0.01	0.08 ± 0.01	0.735 [0.444;0.874] *
Flat Foot Time (s)	0.42 ± 0.09	0.44 ± 0.11	0.940 [0.874;0.971] *	0.29 ± 0.04	0.29 ± 0.02	0.708 [0.388;0.861] +	0.23 ± 0.04	0.24 ± 0.03	0.834 [0.652; 0.921] *
Propulsion Time (s)	0.29 ± 0.05	0.29 ± 0.04	0.834 [0.650;0.921] *	0.25 ± 0.03	0.25 ± 0.02	0.734 [0.442;0.874] *	0.24 ± 0.03	0.23 ± 0.02	0.673 [0.312;0.844] *
Double Support Time (s)	0.36 ± 0.09	0.35 ± 0.09	0.992 [0.982;0.996] *	0.22 ± 0.02	0.21 ± 0.02	0.874 [0.743;0.937] *	0.17 ± 0.03	0.15 ± 0.02	0.932 [0.856;0.967] *
Swing Time (s)	0.48 ± 0.05	0.49 ± 0.05	0.993 [0.986; 0.997] *	0.42 ± 0.01	0.43 ± 0.02	0.939 [0.871;0.971] *	0.39 ± 0.02	0.40 ± 0.02	0.954 [0.903;0.978] *
Foot Progression Angle (°)	−1.38 ± 4.55	−5.56 ± 4.45	0.927 [0.847;0.965] *	−1.74 ± 2.60	−5.25 ± 2.74	0.920 [0.831;0.962] *	−1.36 ± 2.43	−4.63 ± 2.41	0.909 [0.808;0.957] *
Swing Width (cm)	3.02 ± 1.01	3.36 ± 1.63	0.884 [0.757;0.945] *	2.82 ± 0.93	3.15 ± 1.45	0.872 [0.730;0.939] *	2.91 ± 1.02	3.25 ± 1.54	0.896 [0.782;0.951] *
Plantar Flexion Foot In (°)	19.1 ± 2.81	20.5 ± 3.36	0.796 [0.571;0.902] *	21.5 ± 2.31	23.7 ± 2.02	0.830 [0.643;0.919] *	24.38 ± 2.58	25.46 ± 2.34	0.796 [0.570;0.903] *
Plantar Flexion Foot Out (°)	−60.8 ± 5.51	−57.4 ± 5.83	0.841 [0.666;0.924] *	−65.63 ± 3.70	−61.7 ± 3.53	0.738 [0.449;0.875] *	−68.73 ± 4.09	−66.32 ± 3.62	0.813 [0.607;0.910] *
Minimum Toe Clearance (cm)	1.50 ± 0.92	1.75 ± 0.92	0.823 [0.628;0.916] *	1.62 ± 1.01	1.80 ± 1.00	0.741 [0.444;0.874] *	1.61 ± 1.04	1.70 ± 1.11	0.743 [0.452;0.879] *

**Table 6:** Mean and STD of gait parameters for both systems for walking on treadmill. ICC and its 95% CI were also indicated. Significant relations were illustrated as follows: * = p < 0.001 and + = p < 0.002. Non-significant relations were illustrated in italics.

	WALKING ON TREADMILL								
	*Slow Speed*			*Comfortable Speed*			*Fast Speed*		
	Mean ± STD		ICC [95% CI]	Mean ± STD		ICC [95% CI]	Mean ± STD		ICC [95% CI]
	*Motion Capture*	*Insoles*		*Motion Capture*	*Insoles*		*Motion Capture*	*Insoles*	
Speed (m.s^−1^)	0.82 ± 0.11	0.84 ± 0.11	0.999 [0.997;0.999] *	1.08 ± 0.11	1.11 ± 0.11	0.998 [0.996;0.999] *	1.62 ± 0.11	1.63 ± 0.10	0.995 [0.990;0.998] *
Cadence (step.min^−1^)	42.89 ± 3.32	42.89 ± 3.33	0.999 [0.999;0.999] *	50.44 ± 2.85	50.37 ± 2.82	0.998 [0.995;0.999] *	60.43 ± 2.76	60.12 ± 2.68	0.993 [0.985;0.997] *
Stride Length (m)	1.08 ± 0.09	1.17 ± 0.10	0.994 [0.986;0.997] *	1.24 ± 0.09	1.32 ± 0.09	0.991 [0.980;0.996] *	1.57 ± 0.09	1.64 ± 0.10	0.967 [0.930;0.984] *
Stride Time (s)	1.41 ± 0.11	1.41 ± 0.11	0.999 [0.999;0.999] *	1.20 ± 0.07	1.20 ± 0.07	0.997 [0.993;0.998] *	1.00 ± 0.05	1.00 ± 0.05	0.992 [0.982;0.996] *
Stance Time (s)	0.93 ± 0.08	0.92 ± 0.08	0.998 [0.996;0.999] *	0.76 ± 0.05	0.75 ± 0.05	0.994 [0.986;0.997] *	0.60 ± 0.03	0.60 ± 0.03	0.987 [0.973;0.994] *
Loading Time (s)	0.16 ± 0.02	0.11 ± 0.01	0.562 [0.080;0.792]	0.13 ± 0.01	0.10 ± 0.01	0.716 [0.403;0.865] +	0.11 ± 0.01	0.09 ± 0.01	0.762 [0.500;0.887] *
Flat Foot Time (s)	0.50 ± 0.06	0.51 ± 0.06	0.876 [0.740;0.941] *	0.38 ± 0.04	0.37 ± 0.04	0.831 [0.645;0.919] *	0.26 ± 0.03	0.25 ± 0.03	0.740 [0.454;0.876] *
Propulsion Time (s)	0.28 ± 0.04	0.30 ± 0.02	0.767 [0.510;0.889] *	0.25 ± 0.03	0.28 ± 0.02	0.678 [0.324;0.847] +	0.23 ± 0.03	0.26 ± 0.02	0.682 [0.332;0.849] +
Double Support Time (s)	0.44 ± 0.06	0.42 ± 0.06	0.986 [0.971;0.993] *	0.32 ± 0.03	0.31 ± 0.03	0.955 [0.904;0.978] *	0.21 ± 0.02	0.20 ± 0.02	0.891 [0.771;0.948] *
Swing Time (s)	0.49 ± 0.03	0.50 ± 0.03	0.988 [0.976;0.994] *	0.44 ± 0.02	0.44 ± 0.02	0.966 [0.928;0.984] *	0.39 ± 0.02	0.40 ± 0.01	0.934 [0.862;0.969] *
Foot Progression Angle (°)	−1.72 ± 2.59	−6.12 ± 2.94	0.935 [0.863;0.969] *	−1.80 ± 2.68	−5.59 ± 2.97	0.935 [0.863;0.969] *	−1.81 ± 2.36	−4.96 ± 2.49	0.911 [0.812;0.957] *
Swing Width (cm)	3.12 ± 1.18	3.32 ± 2.00	0.899 [0.789;0.952] *	2.97 ± 0.84	3.30 ± 1.38	0.876 [0.738;0.941] *	2.96 ± 0.79	2.97 ± 1.37	0.801 [0.582;0.905] *
Plantar Flexion Foot In (°)	16.48 ± 1.65	16.56 ± 2.14	0.823 [0.629;0.916] *	18.18 ± 2.11	19.14 ± 2.37	0.875 [0.737;0.941] *	22.84 ± 2.47	23.76 ± 1.94	0.855 [0.695;0.931] *
Plantar Flexion Foot Out (°)	−57.01 ± 3.66	−54.61 ± 3.80	0.895 [0.779;0.950] *	−62.55 ± 3.52	−59.45 ± 3.67	0.853 [0.692;0.930] *	−69.45 ± 3.52	−67.43 ± 3.72	0.770 [0.516;0.890] *
Minimum Toe Clearance (cm)	1.74 ± 0.57	1.52 ± 0.80	0.805 [0.589;0.907] *	1.98 ± 0.67	1.64 ± 0.93	0.878 [0.744;0.942] *	1.78 ± 0.67	1.34 ± 0.92	0.814 [0.609;0.911] *

## Data Availability

Data available on request due to restrictions (eg privacy and ethical).

## Supplementary Materials

The following supporting information can be downloaded at: https://www.mdpi.com/article/10.3390/s23198155/s1. References [36,37,38] are cited in Supplementary Materials.
