# Supplementary material for: The Use of Embedded IMU Insoles to Assess Gait Parameters: A Validation and Test-Retest Reliability Study
Source: Sensors (Basel). 2023 Sep 28;23(19):8155. doi: 10.3390/s23198155 (PMC10575241; doi:10.3390/s23198155)
Supplement: Supplementary file 1 [file sensors-23-08155-s001.zip › Sensors_Supplementary_lastVersion_read.pdf]

Supplemantary Data

Table S1. Gait parameters calculation for motion capture system.

Parameters	Calculation and extraction in motion capture
Walking Speed ($m.s^{-1}$)	_Walkway:_ antero-posterior speed of the estimated center of mass (calculated with the resultant of the 4 pelvis markers: RASI, LASI, RPSI and LPSI).
	Treadmill: Absolute average of the minimal antero-posterior velocity of each heel markers (R/LHEE) plus the root mean square (RMS) of the velocity of each heel markers [36] .
Cadence ($step.min^{-1}$)	Step frequency per minute (60 / step time / 2).
Stride Length (m)	_Walkway:_ Resultant of the antero-posterior and mediolateral distances between the heel marker at the initial HS and the heel marker at the following HS.
	Treadmill: Sum of the first step length (antero-posterior distance between the two heel markers at time of contralateral HS) and the second step length (antero-posterior distance between the two heel markers at time of the final HS) [37]. For each step, we added to the step length the push-off length, which is an estimation of the antero-posterior distance traveled by the heel marker (R/LHEE) if the foot was flat on the ground before the rotation of the ankle [38].
Stride Time (s)	Time between initial HS and final HS of the same side.
Stance Time (s)	Time between initial HS and TO of the same side.
Loading Time (s)	Time between initial HS and FFI of the same side.
Flat Foot Time (s)	Time between FFI and FFO of the same side.
Propulsion Time (s)	Time between FFO and TO of the same side.
Double Support Time (s)	Sum of double support times (DS1 + DS2).<br>DS1 = Time between initial ipsilateral HS and contralateral TO.<br>DS2 = Time between contralateral HS and ipsilateral TO.
Swing Time (s)	Time between TO and final HS of the same side.
Foot Progression Angle (°)	Average angle between the foot vector (projected into the laboratory's transverse plane) and the sagittal laboratory axis between FFI and FFO.
Swing Width (cm)	Maximal orthogonal distance between the toe marker (L/RTOE) and this marker projected on the swing line (i.e. line between the position of the toe marker at two different times: TO and final HS).
Plantar Flexion Foot In (°)	Angle between the foot vector (projected into the laboratory's transverse plane) and the transverse laboratory axis at the time of initial HS.
Plantar Flexion Foot Out (°)	Angle between the foot vector (projected into the laboratory's transverse plane) and the transverse laboratory axis at the time of TO.
Minimum Toe Clearance (cm)	Difference between minimum peak vertical displacement during the swing phase and toe height at the toe off [32].

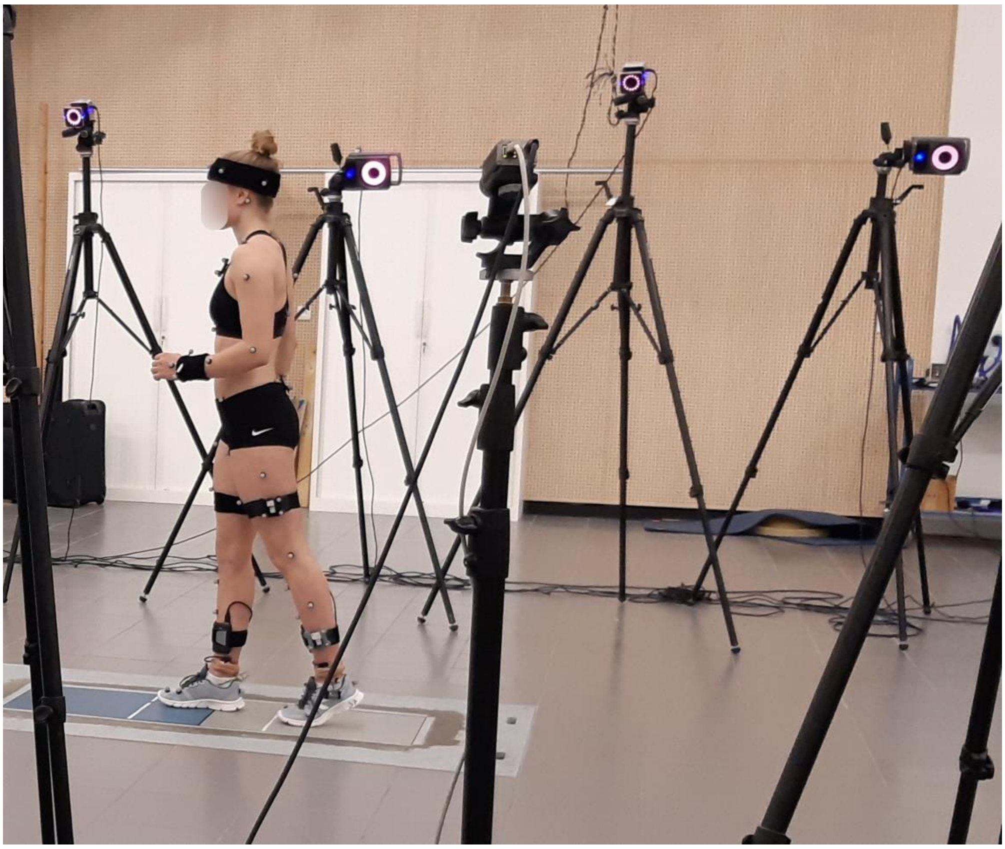

Figure S1. Representation of participant walking along a straight line at their comfortable and self-selected speed.

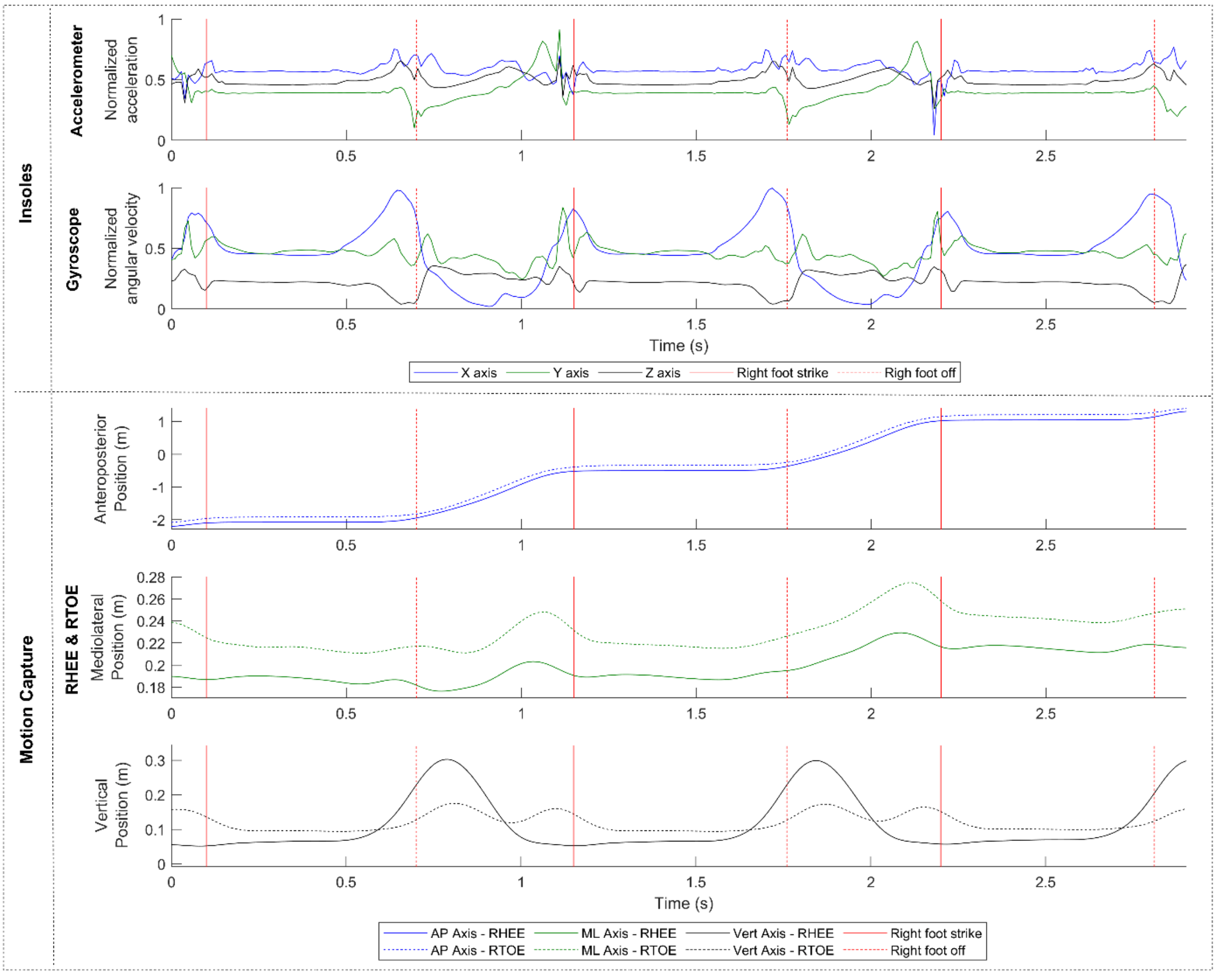

Figure S2. Examples of raw data for motion capture and insoles systems. Blue line = anteroposterior (AP) axis, green line = mediolateral (ML) axis, black line = vertical (Vert) axis, RHEE (Right Heel marker) = solid lines; RTOE (Right toe) = dashed lines, solid red line = Right foot strike, dashed red line = right foot off.

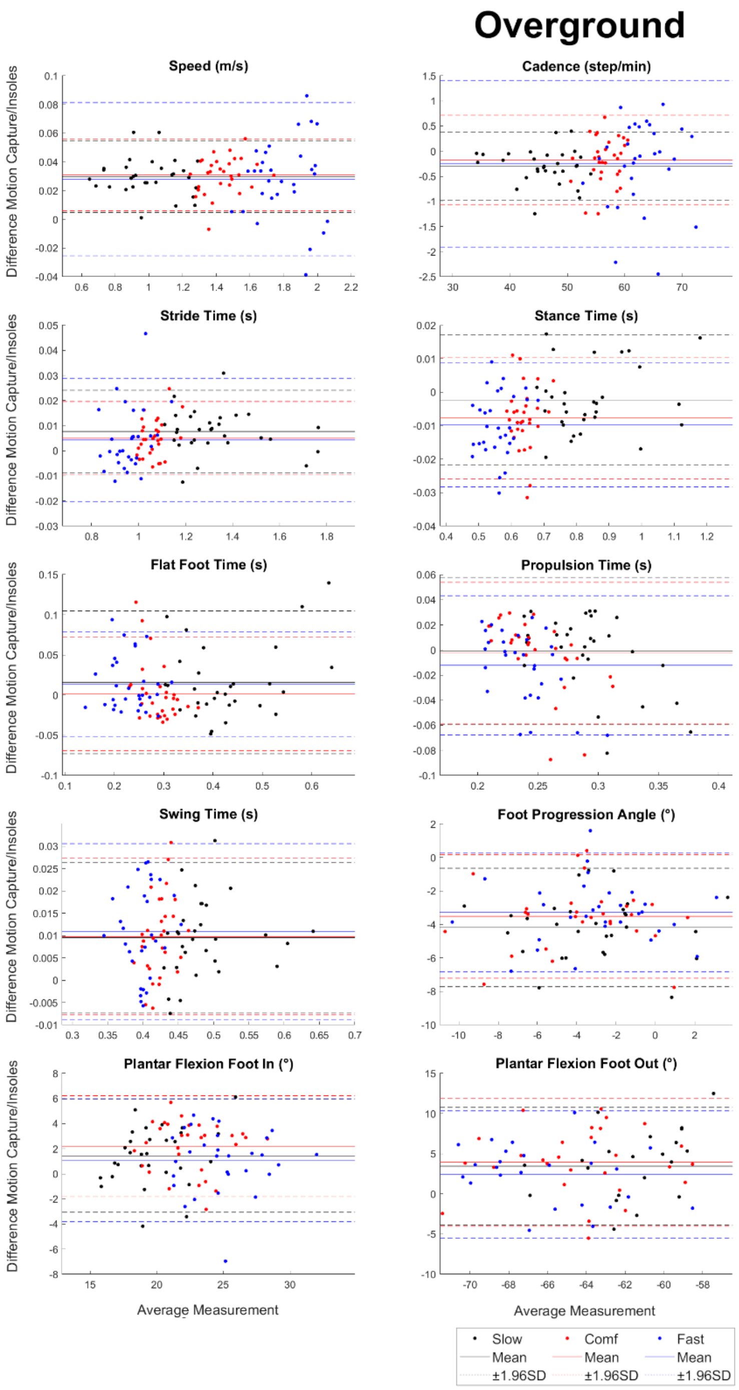

Figure S3. Bland-Altman plot for all gait parameters during fast (blue points), comfortable (red points) and slow (black points) overground walking. Solid line = mean, dashed line = ± 1.96 SD.

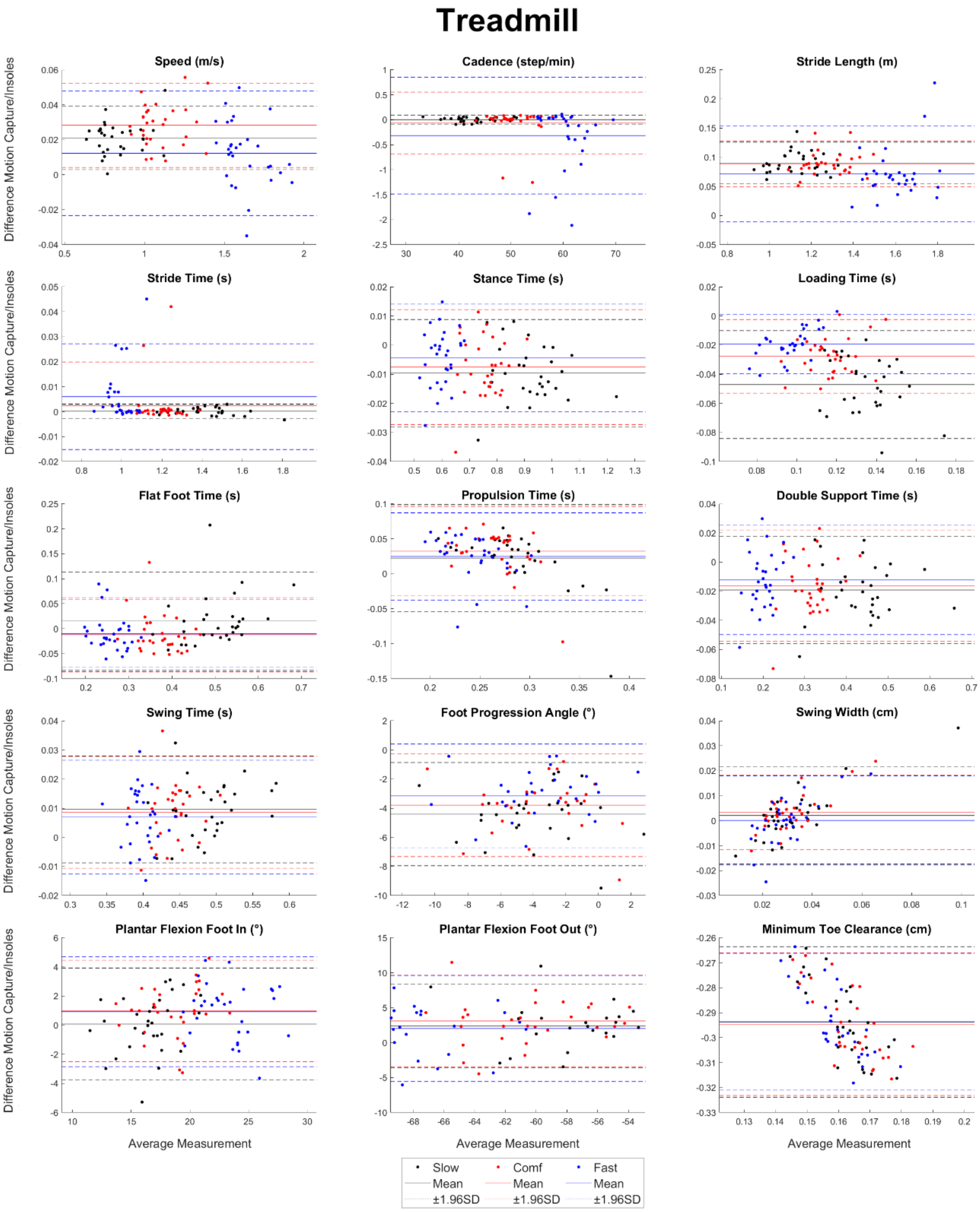

Figure S4. Bland-Altman plot for all gait parameters during fast (blue points), comfortable (red points) and slow (black points) treadmill walking. Solid line = mean, dashed line = ± 1.96 SD.
